# Important Dimensions in Artificial Teeth

**Published:** 1910-03-15

**Authors:** George W. Clapp

**Affiliations:** New York


### IMPORTANT DIMENSIONS IN ARTIFICIAL TEETH.

GEORGE W. CLAPP, D.D.S., NEW YORK.

How shall we select teeth that will set in right relations to the ridge without the need of grinding? This is one of the most difficult questions the plate worker has had to settle. The necessity for getting this dimension right explains why, in almost every dental depot, one sees dentists trying teeth on models; this is the slowest, most inaccurate and most expensive form of tooth selection, but is made necessary by the fact that most dentists do not know how to select a mould of teeth that will set in right relations to the ridge, and bring the cutting edge and neck in the right locations.

Since it is with artificial teeth we are to work, let us study them for a moment and learn what is required that we may select them intelligently. An artificial upper central presents two surfaces which are important for widely different reasons. Note first the labial surface. It is the art surface of the tooth, "the patient's surface," we might call it. Its length from neck to cutting edge, its width from side to side, its outline, and contour are important as to whether or not they harmonize with the patient's face. This is the surface which the patient sees; it largely determines the appearance of the finished plate. It has very little to do with the mechanical side of plate making or with the patient's success in masticating with the plate. But the other surface of the tooth, the overlooked, neglected, little-understood lingual surface, is especially and wholly the dentist's surface. It is the surface whose divisions determine the mechanical fitness of any tooth for the case. It is with this surface that dentists have unconsciously wrestled in the past. This surface, properly understood and utilized, will do much to make the mechanical side of plate work easy. It is this surface, also, which largely affects the patient's success in mastication and clearness of enunciation.

The lingual surface of an anterior tooth shows three divisions. The first begins at the cutting edge and extends cervically to a pronounced ledge; this portion of the tooth is known as "the bite," because it is engaged in biting food by contact with the opposing teeth. The next division presents a flat surface from which the pins project. It is known as "the shut." The third portion extends from "the shut" to the cervical end of the tooth; it is known as "the ridgelap," because it is the only part of the tooth which should lap the ridge.

Dentists have been accustomed to consider these divisions of the lingual surface separately. They have attached much importance to the bite, have practically ignored the shut, and have selected the ridgelap because it was thick or thin linguo-labially. This is not the form of consideration which brings best results. The divisions of the lingual surface of an artificial tooth fall naturally into two classes, according to their functions. The *bite and shut together* form one group because their combined length should carry the incisal edge of the tooth from the surface of the ridge to the labio-incisal angle of the wax bite. Note that it is not the bite of the artificial tooth alone which carries the tooth down; it is the combined bite and shut. Learning first to group these two divisions to meet this requirement will greatly aid any plate worker in tooth selection. The remaining division of the lingual surface, the ridgelap, stands alone as to function. It is supposed to lap the gum or ridge, and to be partially covered by the vulcanite of the plate.

The manufacturer supposes that the dentist will select a mould of teeth in which only the ridgelap will rest against the ridge, and the bite and shut will come below it in an upper, or above it in a lower. To facilitate such selection he offers teeth of the same length and width, with the divisions of the lingual surface in different proportions. For instance, we may have four upper centrals which are of precisely the same length, but in which the divisions of the *lingual* surfaces differ greatly. Not only are the labial surfaces of these centrals of the same length and width, but the six anterior teeth in each of these moulds are of the same combined width, so that, but for differences in the *outlines* of the teeth, plates made from these four moulds appear much alike when seen from the labial surface. But when the teeth are examined from the lingual surfaces, it is seen that hardly any two of them are mechanically suited for the same case. Their lingual surfaces are of the same dimensions in outline, but the length of the bite, shut and ridgelap varies in each tooth. In order that each of these teeth may be properly utilized it should be adapted to the peculiar requirements of the case. The cutting edges of the centrals should come to the labio-incisal angle of the wax bite, and the necks to the high-line, and the bite and shut set properly below the ridge. If the distance between the ridge and the labio-incisal angle of the bite is short, a tooth having a short *combined bite and shut* must be selected if it is to set properly against the ridge. Note that we require not a short bite *only*. Many dentists have given much attention to the length of the bite, entirely overlooking the shut. Here is where they fail. *It is not the length of bite alone that determines the mechanical fitness of a mould; it is the length of the combined bite and shut.* If these, taken together, are not too great, the length of bite may be nearly as desired.

If the distance from the surface of the ridge to the labio-incisal angle of the bite is greater, a tooth with a longer combined bite and shut is required.

When absorption of the alveolar process is practically compete. The distance from the surface of the ridge to the labio-incisal angle of the bite is great; and a tooth having a long combined bite and shut should be used. When the tooth chances to have a moderately long bite, the bite may be either shorter or longer, so that the *combined bite and shut* make up the length required.

If a long ridgelap tooth be used where a long shut and bite are indicated much thicker rubber over the ridge is necessitated and makes the plate more heating to the tissues, and more likely to vulcanize porously. It also interferes with enunciation in speaking and makes proper occlusion difficult.

Plates made with anteriors having at least medium long bites (a medium bite is 3 to 3½ millimeters long) are much less likely to be thrown down behind in the act of biting. It is therefore advisable to use teeth with medium long bites whenever the required length of combined bite and shut makes it possible, save, of course, in end-to-end bite cases.

Selecting teeth with proper bites is very easily done, once the proper length of the *combined bite and shut* has been determined. It is necessary only to decide how much of that distance shall be in the bite. Suppose that the distance from the surface of the ridge is 7½ millimeters, the combined bite and shut of the required artificial central should not be greater than that distance. We may now decide that our bite shall be short (2 to 3 millimeters) or medium (3 to 3½ millimeters) or long (over 3½ millimeters). We may thus decide, before we have looked at a tooth, what general form its lingual as well as its labial surface should have.

Having learned that the bite and shut of an artificial central should set between the surface of the ridge and the labio-incisal angle of the bite, it remains only for us to learn how great that distance is in the case for which we are selecting teeth, and then choose a mould of tooth having a combined bite and shut equal to the distance.

The distance between the surface of the ridge and the labio-incisal angle of the bite is easily learned. Hold the bite so that the surface of the wax which presses against the ridge in the median line can be seen. This can be done by looking into the palatine surface of the bite. Thrust a pin horizontally at the median line from the labial surface of the bite, at such level that it pierces the wax just where the surface of the ridge was.

The pin-hole locates the surface of the ridge on the labial surface of the bite. The distance from the pin-hole to the labio-incisal angle of the bite is the space available for the bite and shut of the central.

It is necessary now only that we learn to reduce the dimensions recorded on the bites to definite measurements, and apply these measurements to the selection of artificial teeth.

We have now seen how to get the distance from the surface of the ridge to the labio-incisal angle of the wax-bite, and how to select teeth having a combined bite and shut that will set them properly to place without grinding. There is one exception to be noted in obtaining this dimension, and, properly observed, it will greatly aid us in selecting teeth, because it affords a wider range of moulds to select from. This exception obtains only in cases where the ridge is thick linguo-labially. It can be best understood by repeating an illustration and studying it from a new view and point.

When the ridge is thin linguo-labially. The pins of the artificial tooth will extend nearly across it, and they will come on the lowest part of its surface. Therefore, in this case, all that has been said above about the combined bite and shut not being greater than from the lowest point of the ridge to the labio-incisal angle applies. But when we have a different type of alveolar ridge, as when it is thick linguo-labially, and the teeth are set as they would be in most cases (the labial surface of the built-up wax-bite will show how they are to set for any given case), the teeth cannot reach far enough under the ridge to bring the bite and shut below *the lowest point*, but they will come anterior to and a little above that point. Therefore, when the ridge is thick linguo-labially and the teeth are to set slightly forward of the ridge instead of right against it, the combined bite and shut may be one or two millimeters greater than the distance between the pin-hole and the labio-incisal angle. And the teeth will still go to place without grinding.—*Dental Digest*, March, 1909.

